# Methicillin-Resistant Staphylococcus aureus Eradication and Decolonization in Children Study (Part 1): Development of a Decolonization Toolkit With Patient and Parent Advisors

**DOI:** 10.2196/14974

**Published:** 2020-05-20

**Authors:** Courtney M Moore, Sarah E Wiehe, Dustin O Lynch, Gina EM Claxton, Matthew P Landman, Aaron E Carroll, Paul I Musey

**Affiliations:** 1 Research Jam Indiana Clinical and Translational Sciences Institute Indianapolis, IN United States; 2 Children's Health Services Research Department of Pediatrics Indiana University School of Medicine Indianapolis, IN United States; 3 Department of Surgery Indiana University School of Medicine Indianapolis, IN United States; 4 Pediatric and Adolescent Comparative Effectiveness Research Department of Pediatrics Indiana University School of Medicine Indianapolis, IN United States; 5 Department of Emergency Medicine Indiana University School of Medicine Indianapolis, IN United States

**Keywords:** Staphylococcus aureus, MRSA, abscess, decolonization, human-centered design, communication design

## Abstract

**Background:**

Community-acquired methicillin-resistant *Staphylococcus aureus* (MRSA) skin and soft tissue infections affect many healthy children. A significant number of these children are hospitalized and require surgical incision and drainage (I&D). Once sent home, these children and families are asked to complete burdensome home decolonization and hygiene procedures in an effort to prevent the high rate of recurrent infections.

**Objective:**

This component of the Methicillin-resistant *Staphylococcus aureus* Eradication and Decolonization in Children (MEDiC) study aimed to develop a toolkit to assist MEDiC study participants in completing MRSA decolonization and hygiene procedures at home (the MEDiC kit).

**Methods:**

In all, 5 adolescents (aged 10-18 years) who had undergone an I&D procedure for a skin infection and 11 parents of children who had undergone an I&D procedure for a skin infection were engaged in a 4-hour group workshop using a human-centered design approach. The topics covered in this workshop and analyzed for this paper were (1) attitudes about MRSA decolonization procedures and (2) barriers to the implementation of MRSA decolonization and hygiene procedures. The team analyzed the audio and artifacts created during the workshop and synthesized their findings to inform the creation of the MEDiC kit.

**Results:**

The workshop activities uncovered barriers to successful completion of the decolonization and hygiene procedures: lack of step-by-step instruction, lack of proper tools in the home, concerns about adverse events, lack of control over some aspects of the hygiene procedures, and general difficulty coordinating all the procedures. Many of these could be addressed as part of the MEDiC kit. In addition, the workshop revealed that effective communication about decolonization would have to address concerns about the effects of bleach, provide detailed information, give reasons for the specific decolonization and hygiene protocol steps, and include step-by-step instructions (preferably through video).

**Conclusions:**

Through direct engagement with patients and families, we were able to better understand how to support families in implementing MRSA decolonization and hygiene protocols. In addition, we were able to better understand how to communicate about MRSA decolonization and hygiene protocols. With this knowledge, we created a robust toolkit that uses patient-driven language and visuals to help support patients and families through the implementation of these protocols.

**Trial Registration:**

ClinicalTrials.gov NCT02127658; https://clinicaltrials.gov/ct2/show/NCT02127658

## Introduction

### Background

The past two decades have seen a dramatic increase in community-acquired skin and soft tissue infections (SSTIs), such as cellulitis, boils, myositis, and abscesses, caused by the antibiotic-resistant bacteria known as methicillin-resistant *Staphylococcus aureus* (MRSA) [1-8]. The shift from hospital-acquired infections to community-acquired infections has resulted in many healthy children being affected [2,4]. Recent estimates indicate that the incidence of hospitalizations in the United States caused by MRSA SSTIs is more than 45 per 100,000 children, with many children requiring surgical procedures such as incision and drainage (I&D) [6,9,10]. The rate of recurrent infection can be as high as 72% [11-16].

As frequent recurrence of MRSA SSTIs is believed to increase suffering, health care utilization, and cost, strategies to decrease the rate of recurrence are necessary. MRSA colonization (presence of bacteria on the skin and in the nose) has been demonstrated to be a risk factor for SSTIs and their recurrence, and there has been a high prevalence of MRSA colonization among patients presenting to emergency departments in the United States [17,18]. Decolonization protocols using topical mupirocin ointment in the nose to eliminate nasal carriage and chlorhexidine or bleach baths to eliminate skin carriage [19-21] are often recommended by the Infectious Disease Society of America as strategies to eradicate the bacteria and decrease the recurrence of hospital-acquired MRSA infections [11]. The prevention of community-acquired MRSA has been studied to a much lesser degree and the success rates of these protocols are mixed [11,15]. Additionally, few studies have addressed the burden of these decolonization protocols that often consist of regular bleach baths or chlorhexidine body washes and daily nasal antibiotics, on patients and their families. One study that aimed to better understand the feasibility of decolonization protocols found that only 38% of participants reported having completed both components of a 5-day protocol involving twice daily mupirocin nasal swabs and once daily diluted bleach baths. Some barriers reported by participants were side effects such as skin irritation, aversion to the smell of bleach, and being too busy [22]. This study sheds some light on barriers that may exist in MRSA decolonization, but is in the context of only a very short 5-day protocol and does not shed light on how these barriers might be addressed.

### Objectives

With the aid of patients and families with lived experiences with MRSA SSTIs, we sought to design the Methicillin-resistant *Staphylococcus aureus* Eradication and Decolonization in Children (MEDiC) comparative effectiveness trial assessing 2 interventions over the course of 12 months: (1) abscess surgery and hygiene education compared with (2) abscess surgery and hygiene education followed by decolonization [23]. Knowing that decolonization procedures can be burdensome, we engaged patients and their families (referred to as advisors herein) in a human-centered design (HCD) workshop to understand (1) their attitudes about MRSA decolonization procedures and (2) uncover potential barriers to the implementation of MRSA decolonization and hygiene procedures. This paper will discuss the workshop activities, results, and how our findings informed the creation of a *toolkit* to assist participants in the MEDiC intervention study with decolonization and hygiene procedures. A companion paper discusses a separate objective of this project, which was to engage patients and their families to better understand what outcomes were important to them when it came to MRSA decolonization and to select measures to capture these as part of the MEDiC study [24].

## Methods

### Overview

This patient engagement project was the first step in designing an MRSA decolonization toolkit to prepare for a randomized comparative effectiveness trial (MEDiC-NCT02127658), as described briefly earlier. This study was approved by the Indiana University School of Medicine Institutional Review Board. Participants were eligible if they were a patient (aged 9-18 years) who had undergone an I&D procedure at Riley Hospital for Children or a parent of a patient over 3 months of age who had undergone an I&D procedure at Riley Hospital for Children. Written informed consent was obtained from all participants over the age of 13 years. Participants aged 9 to 13 years provided assent. All participants received US $20 per hour for the 4-hour audio-recorded workshop.

### Human-Centered Design

The Indiana Clinical and Translational Sciences Institute’s patient engagement core, known as Research Jam (RJ), uses an HCD approach to co-design better research study experiences with study stakeholders. HCD developed out of the ergonomics and computer science [25] fields and has expanded widely to be used in the creation of a vast array of products, services, and experiences across many different fields. HCD is a qualitative approach to understanding human needs, designing solutions that address these needs, and doing so hand in hand with stakeholders throughout the process. Stakeholders are considered experts in the problem area and are engaged as advisors and co-designers in the HCD process. The involvement of stakeholders is generative (allowing them to create the possibilities) rather than solely evaluative (allowing them to respond to predefined possibilities). RJ collaborated with the principal investigator (PM) to explore potential barriers the kit would need to overcome as well as desired messages (the framing of the information) and media (the format through which the information is delivered) and apply this knowledge to create the MEDiC kit. The workshop, facilitated by HCD experts DL and CM, and using the principles of HCD, comprised the following 3 methods.

### Method I: Media Warm-Up

To begin, the participants (who will be referred to as advisors) were each asked to share their name and two ways they like to get information. The answers to the latter were written on a flip chart. This accomplished 2 goals: (1) to allow the advisors to get acquainted with one another and comfortable speaking aloud to the group and (2) to understand the preferred method of obtaining information, setting a foundation for further discussion.

### Method II: Task Analysis of Methicillin-Resistant Staphylococcus Aureus Decolonization and Hygiene

The team used a simplified version of hierarchical task analysis, which examines the tasks needed to achieve a goal, ultimately informing strategies that enable effective use of systems or products. Task analysis is often used to iterate operations within a system, frequently resulting in changes to the tasks themselves [26]. In our case, the tasks to be analyzed are an accepted protocol, and so our focus was not on changing the tasks but changing the artifacts designed to support the tasks and to support behavioral changes. Rather than diving deeply into each task as is possible with task analysis, we chose to focus on breadth across the varied tasks in hopes to better understand the challenges posed by the broader personal, family, and environmental contexts in which these tasks might be undertaken. In this way, we hoped to create support artifacts that respond to these challenges and help families adhere more successfully to the study protocol. We separated the MEDiC study intervention goal of MRSA decolonization into 12 proposed tasks:

1. Uniforms and practice jerseys should be washed after each game or practice. Other sports equipment should be cleaned weekly.
2. Do not share towels, washcloths, clothing, toothbrushes, or razors with family or friends.
3. Wash all towels, washcloths, sleepwear, underwear, and linens in detergent and hot water once weekly and dry hot in a dryer.
4. Take daily showers or baths with soap.
5. Shower before and after all sports practices and competitions and wipe down all equipment before and after use.
6. Clean hands with soap and water (or hand sanitizer) when hands are dirty and after bathroom breaks and diaper changes.
7. Apply 2% mupirocin ointment to nostrils using a cotton swab twice a day for 10 days.
8. Twice weekly for 6 weeks, take a bath for 15 min in diluted bleach water.
9. Diluted bleach water should be made with 1 cup of 6% sodium hypochlorite bleach for a 50-gallon bathtub of water or 1 tsp per gallon of water.
10. Keep all wounds, including cuts and scrapes, clean and covered until healed.
11. Avoid other people’s dirty bandages or uncovered wounds.
12. Throw away all lotions in jars.

Advisors were asked to review the tasks and identify pain points—potential moments of difficulty—and brainstorm ways to overcome these pain points. Each task was displayed on a flipchart. Advisors were asked *What might make it hard to do this?* and responded by writing on individual post-it notes as many responses as they could think of. For the first round of responses, the advisors were asked to individually write their answers on post-it notes and add these post-its to the tasks to which they referred. For the second round of brainstorming, facilitators (CM and DL) read the answers aloud to the group and asked follow-up questions about the content of the notes to clarify meaning and encourage discussion to uncover additional pain points. Once the advisors had no other pain points to contribute, the facilitator asked advisors to brainstorm ways to overcome the major pain points, and these were added to the flipchart pages. The facilitator referred advisors to the flipchart from the media warm-up containing ways they liked to get information to help prompt additional ideas.

### Method III: Five Senses Maps

A Five Senses map is used to display sensory information that a particular subject evokes. Typically, the subject is a brand, and the sensory information is aspirational—senses the brand should evoke with its customers through all aspects of its interactions with them, called sensory marketing [27]. In the context of this project, the Five Senses map visualization was used as a tool to structure our questioning, keep responses visible to the advisors, and allow the advisors to collaboratively build early maps during the workshop that would serve as prototypes for later maps. The goal of this 3-part activity was to understand the advisors’ sensory experience of bleach, both positive and negative. Understanding the perceptual barriers to accepting and following a bleach bath protocol would allow the team to craft messaging or make adjustments to the protocol to help overcome these perceptions. The first part of the activity asked the advisors—1 sense at a time—to talk about the smells, sounds, tastes, sights, and tactile feelings bleach brought to mind. These were written by a facilitator CM on a flip chart in the form of a Five Senses map. The second part of the activity was to look at the senses captured on the Five Senses map for bleach and suggest opposite senses to create a new Five Senses map that represents the exact opposite of bleach. This helped the team further interpret and define what the advisors meant by the senses they associated with bleach. The last part of this activity asked the advisors to think about how they might change bleach to make it more like the opposite Five Senses map. These answers were captured on a flipchart.

### Analysis

**Figure 1:**
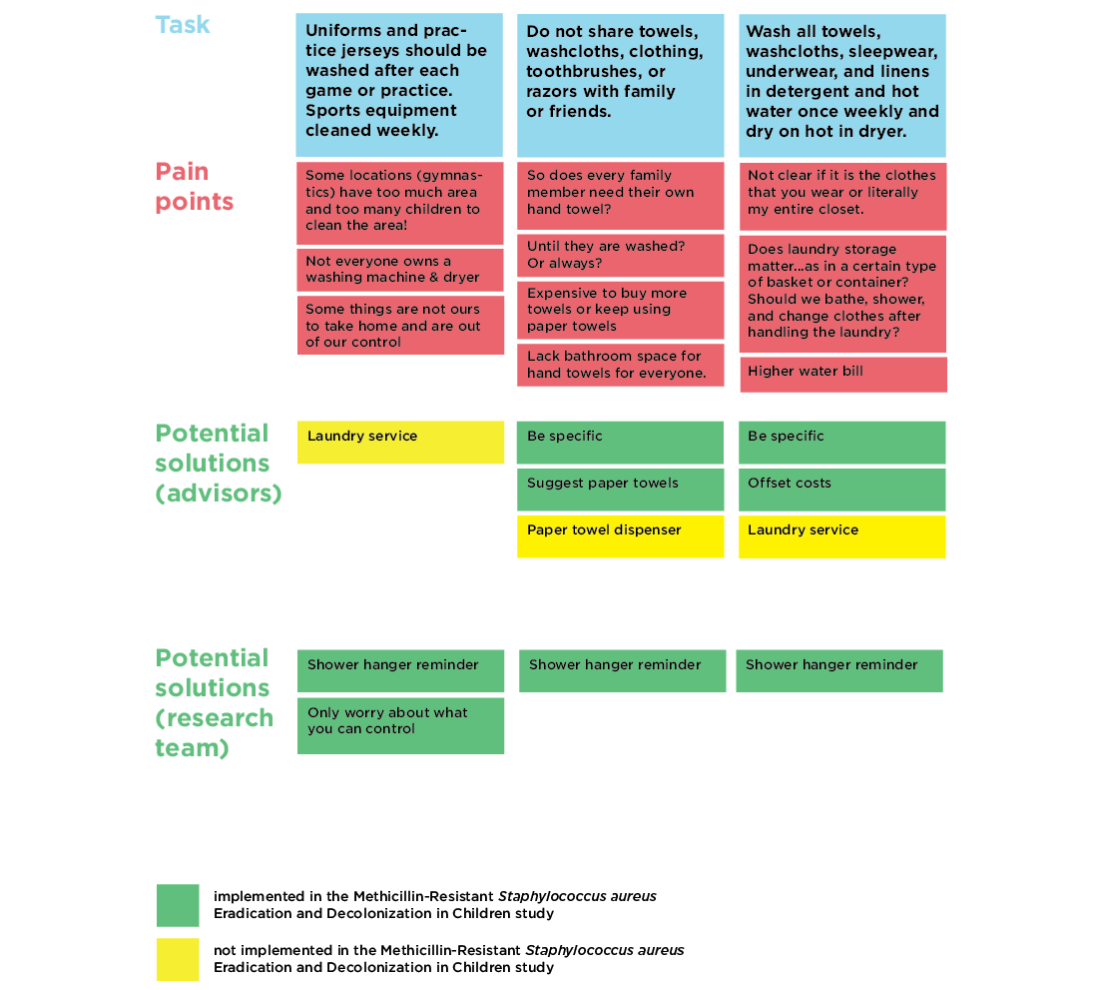
Task analysis grid 1.

To prepare for analysis, the audio related to the Five Senses mapping activity was reviewed by CM and DL, and information not captured on the Five Senses maps was added to the maps. The task analysis responses were transcribed by CM into individual snippets of information. Audio related to the task analysis activity was also reviewed, and content missing from the written responses was transcribed by CM onto individual snippets of information. The data, information, knowledge, wisdom (DIKW) framework, popularized by Ackoff and expanded upon by Kolko, guided the analysis process for the workshop data. DIKW is a framework that organizes the evolution of findings from data (discrete symbols) to wisdom (development of increased value through the application of knowledge) [28,29]. Team members CM and DL were guided by Kolko’s synthesis methods for jumping the gaps between each stage of DIKW [29]. Through affinity clustering, the snippets of data were examined to find similarity in meaning and then grouped into clusters to begin to organize the data to create meaning. These meaningful data are what Kolko defines as information. The information clusters were given names to capture the meaning represented by the data they contained. Next, CM and DL engaged in visual modeling, which Kolko suggests for moving from information to knowledge (principles, theories, or stories) and involves organizing the clusters from the previous step into visual structures that present hypotheses about their relationships with each other. These visual models were (1) a task analysis grid (a model of tasks, pain points, and potential solutions from the advisors) and (2) a final Five Senses map that captured the positive associations of bleach while shifting the negative associations. The team (CM and DL) then reviewed the affinity diagrams and visual models and identified insights or key patterns that, when combined with new knowledge developed through the analysis process, had implications for the design product (the MEDiC kit). On the basis of these insights and existing expertise and experience, the team (CM, DL, and PM) identified additional potential solutions to add to the task analysis grid (Figure 1) and created the design of the final MEDiC kit. This application of knowledge is defined by Kolko as wisdom.

## Results

### Workshop Participation

We engaged a total of 16 workshop advisors, including 5 adolescents (3 females aged 10, 14, and 18 years; 2 males aged 14 and 17 years), all of whom had undergone an I&D procedure in addition to 11 total parents (10 females and 1 male); 4 of these were parents of the adolescents in attendance, and the other 7 were parents of younger children (aged 15 months to 5 years) who had also undergone an I&D procedure. Parents and their adolescent children and parents of younger children were split into separate small groups during the task analysis portion of the workshop. Adolescents remained in the room with their parents during the workshop.

### Key Pain Points

Using the methodology described above, the following barriers, or *pain points*, were identified by the advisors in response to the 12 tasks proposed to help prevent MRSA recurrence. Figure 1 shows an example of the task analysis grid, which organizes each of the tasks in the decolonization and hygiene protocols and their associated pain points (as identified by the advisors). Potential pain point solutions offered by the advisors are also captured on the grid. The solutions in green were implemented as part of the kit, whereas those in yellow were deemed to be not feasible or not within the scope of this project (see Multimedia Appendices 1-3 for the remainder of the task analysis grid). This section discusses the key barriers we heard from the advisors as well as what we did to address each in the kit.

### Current Decolonization Instructions Are Not User Friendly and Do Not Answer Advisors’ Questions

#### What We Heard

Some of the advisors were already using a bleach bath protocol recommended by a physician. They were given the majority of the instructions verbally, although some advisors had been given brief written instructions. The instructions were not consistent across the advisors. A few of the families reported that they had watched videos on YouTube that had been created by other parents to show the process step by step. They found these helpful and more user-friendly than instructions they had been given by their physicians, but they were unsure whether or not they could trust the information presented because it was not from a physician. They suggested a video of families going through a decolonization protocol step by step created in partnership with a doctor or another trusted source. During the warm-up activity, advisors discussed their preferred methods of obtaining new information. Video was the most popular method. Learning by doing (hands-on) was the second most popular. Other methods that were mentioned included being trained, getting notifications, reading instructions, drawing out a plan, getting text messages, and looking into all the options before choosing one for implementation.

For some parts of the protocol, the draft instructions presented to the advisors left too much to interpretation. For example, the advisors questioned whether or not they needed to wash all the clothing and linens they owned once a week or just those they had used. Additionally, they wondered if laundry should be stored and handled in a particular way and if they should shower after handling dirty laundry. Other similar questions were as follows:

1. Can 1 cotton swab be used for applying the ointment to the nostrils (1 side per nostril) or should 2 different cotton swabs be used?
2. Does everyone in the family need their own hand towels?
3. Can towels be shared after they are washed or does 1 towel have to always belong to the same person forever?
4. Can you never ever use jars of lotion, or is it just that we need to start fresh and spoon it out from now on?

The advisors wanted to understand what the instructions in the protocol were accomplishing. Discarding lotion in jars was one part of the protocol that inspired many comments. For some of the advisors, this would mean throwing a lot of money in the trash, so they wanted a clear explanation of why this was necessary. Once the reason was explained, the advisors understood, but without a clear reason, this just seemed wasteful.

#### What We Did

Through collaboration between content expert PM and communication designers DL and CM, the team developed detailed, user-friendly instructional materials (Multimedia Appendix 4) for hygiene and decolonization that responded to the informational needs and questions expressed by the advisors.

Because the advisors preferred visual methods of getting new information, the team created visuals to accompany the written instructions. In addition, the team created a step by step instructional video featuring a medical professional (AC; Multimedia Appendix 5).

The final kit includes much of the information advisors asked for during the task analysis discussion to help them better understand and adhere to the procedures. For some of the procedures, this included being more explicit (eg, specifying that only used clothing and linens ought to be washed once a week). This also included giving reasons for adhering to procedures with less obvious justification (eg, that lotions in jars are easily contaminated and that is why they ought to be avoided).

### Families May Not Have the Proper Tools for Implementing Decolonization and Hygiene Procedures

#### What We Heard

Bleach packaging makes it difficult for families to implement decolonization protocols as recommended. Bleach comes in heavy bottles with large openings for pouring. This makes it very difficult to pour a small amount of bleach into a small measuring cup (assuming you have one on hand) to obtain the proper measure of bleach. To overcome this, the advisors suggested having a pour spout that slows down the flow of bleach, having a pump that dispenses a consistent amount of bleach each time, or using bleach tablets that you can drop into the bath. Families also may not know the volume of their bathtub and may not have an empty gallon jug on hand to measure it, which might force them to guess and have an unknown and inaccurate bleach-to-water ratio.

#### What We Did

To ensure that the MEDiC study participants had the right equipment to complete the study procedures, they were provided with a bucket, a large measuring cup, a small measuring cup to use for infant baths, bleach, wax crayon, mupirocin, and cotton swabs in addition to the printed instructional materials in the MEDiC kit. *Splash-less* bleach was chosen because it has a thicker consistency and can be poured more easily. A packing slip (Multimedia Appendix 6) included a visual list of all of the items study participants were intended to receive.

### Some Tasks Were Out of the Control of Families

#### What We Heard

The advisors discussed the difficulty of adhering to some parts of the protocol that may not be within their control. This is particularly true of those parts of the protocol that involve gyms or sports equipment. In some sports, the area is too large to reasonably expect a family to wipe down (eg, gymnastics mats and floors). In other cases, sports equipment might not belong solely to the athlete and might be managed by another person, making it impossible for families to clean or launder them. In other cases—such as at a gym—the correct supplies may not be available for cleaning off equipment. In addition, children are often under the care of school staff during much of the day, making it difficult for caregivers to ensure that they are washing their hands properly, not removing bandages, not sharing objects with other children, etc. Additionally, because small children need help with hygiene practices such as applying bandages, it may be difficult for a caregiver to avoid a child’s wounds. In other cases, children may be under the care of another parent in a different household, which might necessitate an extra set of all of the supplies and instructions and buy-in from the other parent. Children may also be under the care of a babysitter in their own home who would need to be properly trained to undergo the various protocol pieces.

#### What We Did

Parents were very concerned about their lack of ability to control some portions of the protocol. It can be challenging to perfectly adhere to procedures that require such careful control inside and outside of the home. The team included content to reassure parents that they did not need to have perfect control, but that they should strive to do their best (eg, to take precautions when helping small children with MRSA with their wounds). For the MEDiC study, the team wanted to ensure participants were adhering to the protocol without causing them unnecessary anxiety and that they were encouraged to be honest when tracking for the study. In addition, materials such as the instructional video could function as a tool for parents to disseminate knowledge to other caregivers such as grandparents and daycare providers as needed.

### Decolonization and Hygiene Tasks Are Burdensome

#### What We Heard

The hygiene protocol requires keeping a lot of information, events, and supplies organized, which the advisors thought might be difficult for some families. Remembering all of the protocol requirements is difficult, and keeping track of which family member has done which part of the protocol on which day makes this all the more complicated. In addition, keeping track of which hand towel, bath towel, washcloth, toothbrush, etc, is whose can be very difficult in a large household with only one bathroom.

#### What We Did

The team created materials to function as reminders for MEDiC study participants. In all, 2 study task tracking booklets were created; one for the hygiene plus decolonization arm of the study and one for the hygiene-only arm (Multimedia Appendices 7 and 8). In addition, a hygiene shower hanger (Multimedia Appendix 9) was created to keep important hygiene tasks visible for all participants. In addition, the team included suggestions that might ease the burden of some of the procedures (eg, suggesting paper towels instead of individual bathroom hand towels for each family member).

### Some of the Advisors Had Incorrect Assumptions About Methicillin-Resistant Staphylococcus Aureus

#### What We Heard

Throughout the workshop, the advisors shared information that they had heard about MRSA. Some advisors thought it was transmitted from parent to child through DNA or blood. Others thought it was a virus. Before the workshop, 2 of the mothers had thought it was only caused by diapers, and once their children were out of diapers, the MRSA would be gone. One mother suspected that her child contracted MRSA from being on the beach in Florida. These responses highlight a lack of basic education about MRSA, even for families experiencing it firsthand.

#### What We Did

As the advisors had misconceptions about MRSA, the team developed a summary of basic information about MRSA to help educate MEDiC study participants. The summary is included in the tracking booklets and includes information about what MRSA is, who is most at risk of MRSA infections, and how MRSA is spread. In addition, the kit’s tracking booklets included answers to common questions the advisors asked about MRSA. In particular, they answer many of the questions about what activities a person with MRSA should avoid during an outbreak, as this was a major concern and knowledge gap for the advisor group.

### Bleach Has Positive and Negative Connotations

#### What We Heard

**Figure 2:**
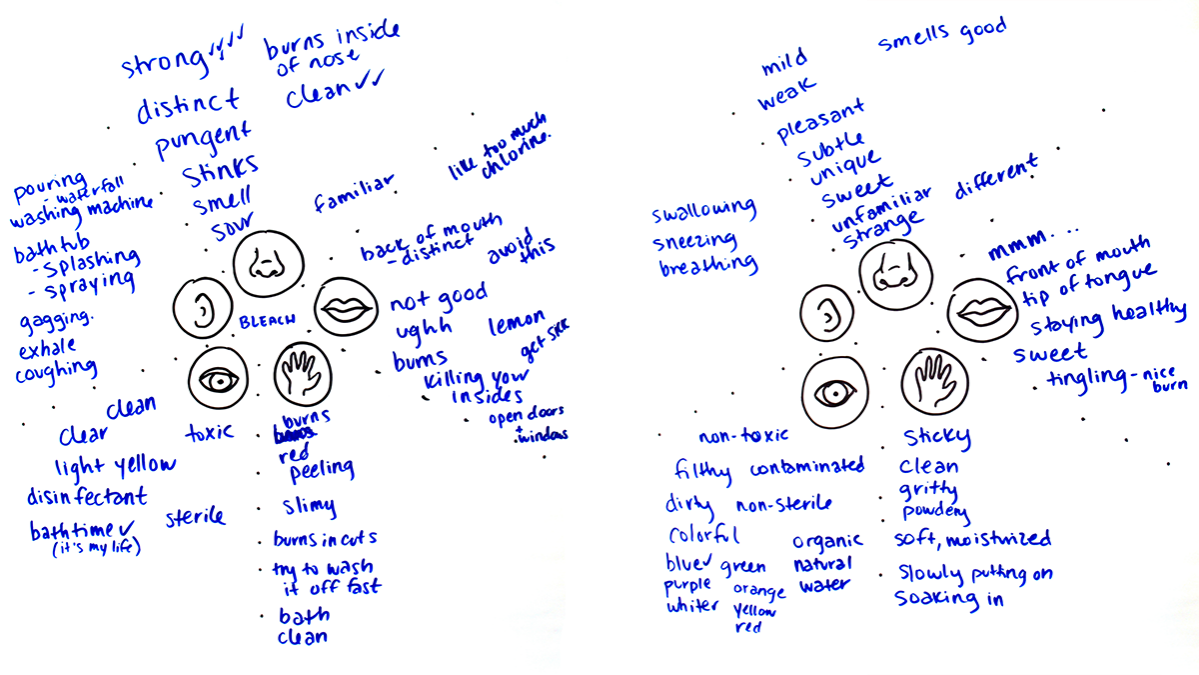
Five Senses map of bleach and the opposite of bleach.

The Five Senses map for bleach (Figure 2) was not entirely negative. Bleach was perceived as clean and sterile, a tool for disinfecting and ensuring a safe environment. Many of the advisors had already utilized bleach bath protocols for their children, so bleach was also associated with familiarity and bath time. This is bolstered by the opposite Five Senses map (Figure 2), which includes words such as “dirty” and “contaminated.” Despite the positive associations, bleach was also associated with many negative senses. It was seen as something that would cause negative effects such as burning the skin, nose, and eyes and causing gagging. One advisor associated the sense of taste of bleach with “killing your insides” because it was toxic to consume. On the opposite Five Senses map, the advisors used words such as “pleasant,” “sweet,” “subtle,” and “tingling” to describe the opposite of bleach.

#### What We Did

**Figure 3:**
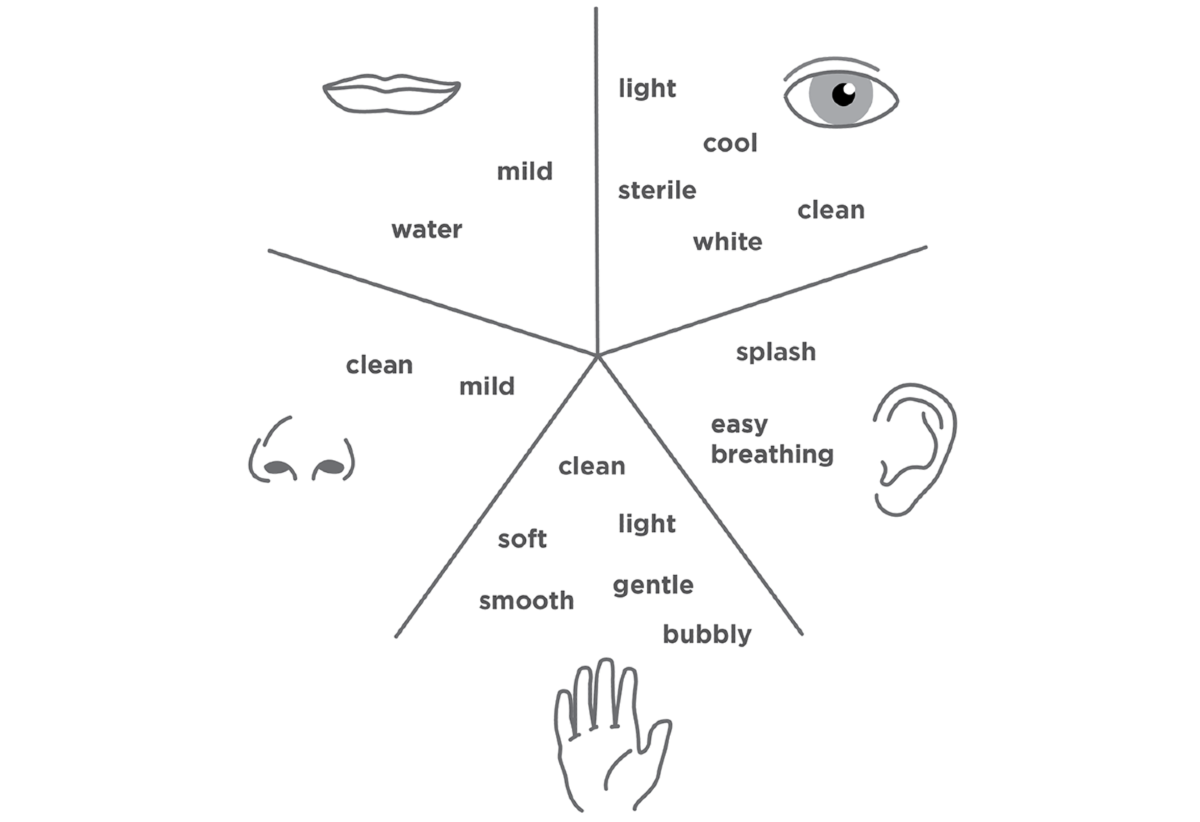
Five Senses map of the Methicillin-Resistant Staphylococcus aureus Eradication and Decolonization in Children study.

Team members DL and CM, who have expertise in visual communication design, created a final Five Senses map for the MEDiC study itself that incorporates the positive aspects of the Five Senses map of bleach and the opposite Five Senses map of bleach (Figure 2). This final map (Figure 3) guided branding and design efforts. The idea of utilizing bleach baths, particularly for children, understandably produces anxiety for some parents. The Five Senses map of bleach shows that bleach is very stimulating; therefore, the goal for visual communication was to provide a more mild, low-impact backdrop for the study by being visually well-organized with low visual stimulation.

**Figure 4:**
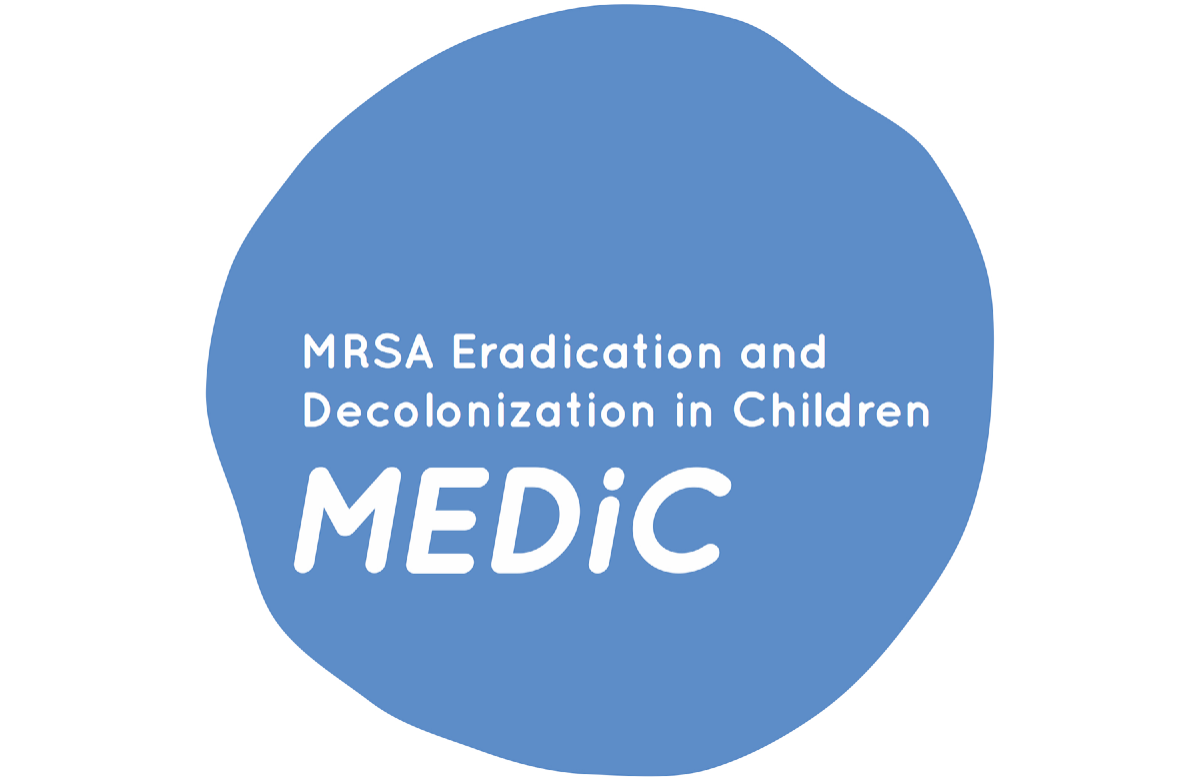
The Methicillin-Resistant Staphylococcus aureus Eradication and Decolonization in Children study logo.

A final logo was created (Figure 4). The logo represents both a water droplet and the shape of MRSA under a microscope and uses cool colors as recommended by the Five Senses map. In addition, an Indiana University logo was included in all of the materials to lend credibility to the content.

The kit uses photographs to show MEDiC study participants what the materials are and how they are used. To keep the designed materials light, clean, soft, etc, the page layout is simple with an abundance of white space. The chosen paper stock is bright white, and the text is gray rather than a visually harsher black. The covers of the tracking booklets are a plain pattern so that MEDiC study participants can carry them without signaling to strangers that they are in an MRSA study.

#### The Final Kit Components

**Figure 5:**
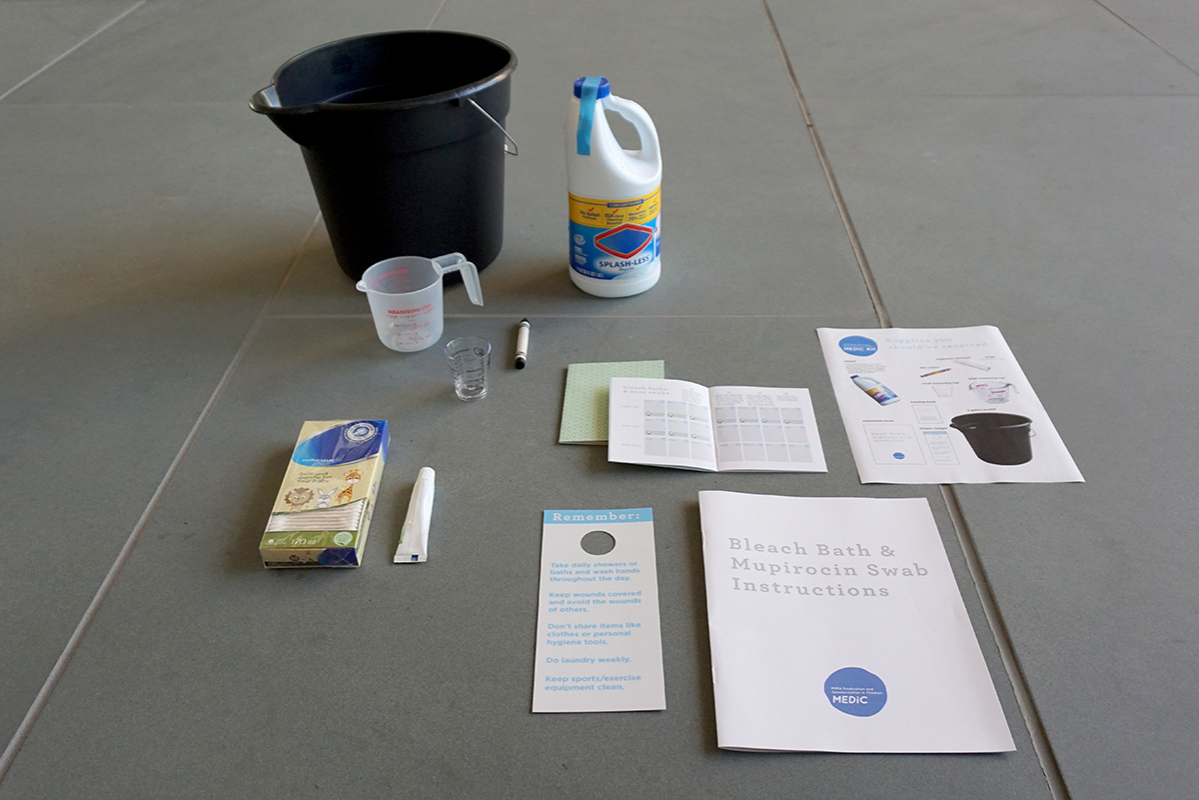
The Methicillin-Resistant Staphylococcus aureus Eradication and Decolonization in Children study kit components.

The following is a summarized list of each of the final components of the MEDiC kit, which can be seen in Figure 5. The MEDiC kit is available for download on the Research Jam website [30]. The MEDiC trial randomizes participants into 2 arms: a decolonization arm and a hygiene-only arm. Some of the kit components were given to one arm or the other or to both. This is indicated in parentheses in the descriptions that follow.

1. *Decolonization instruction booklet (decolonization*; Multimedia Appendix 4): This booklet contains detailed step-by-step photographic instructions for preparing and completing bleach baths and mupirocin swabs and tracking them with the decolonization tracking booklet. The instruction booklet also includes a link to the bleach bath instructional video on the Web.
2. *Decolonization instructional video (decolonization*; Multimedia Appendix 5): This is a short video showing the same step-by-step information included in the decolonization instruction booklet for both the bleach baths and the mupirocin swabs. The demonstration was performed by a pediatrician from Riley Hospital for Children (AC) to ensure that the MEDiC study participants know the information is reliable.
3. *Decolonization tracking booklet (decolonization*; Multimedia Appendix 7): A booklet to help families who are randomized into the decolonization group track hygiene and decolonization tasks as well as MRSA-related outcomes. The book begins with information about MRSA to help patients and families understand better how MRSA happens and to answer key questions advisors in our group asked. This tool aids patients and their families in keeping track of the MRSA hygiene and decolonization tasks they complete, making it easier for MEDiC study participants and more accurate for researchers.
4. *Packing slip (decolonization*; Multimedia Appendix 6): A photographic list of decolonization supplies sent to families randomized to the decolonization group. The supplies include a 3-gallon bucket, a wax crayon, a small measuring cup, a large measuring cup, *Splash-less* bleach, cotton swabs, mupirocin, a hygiene shower hanger, a decolonization tracking booklet, and a decolonization instruction booklet.
5. *Hygiene shower hanger (both*; Multimedia Appendix 9): A shower hanger with reminders for good MRSA hygiene (to be printed and laminated before use). This helps patients and families remember key things they should be doing for the hygiene portion of the protocol and is located in a place where they will see it every day: the shower.
6. *Hygiene tracking booklet (hygiene only*; Multimedia Appendix 8): A booklet to help families who are randomized into the hygiene-only group track MRSA hygiene tasks and MRSA-related outcomes. The book begins with information about MRSA to help patients and families understand better how MRSA happens and to answer key questions advisors in our group asked. This tool aids patients and their families in keeping track of the MRSA hygiene activities they complete, making it easier for MEDiC study participants and more accurate for researchers.
7. *Decolonization supplies (decolonization*; Multimedia Appendix 4 gives more information on how the supplies are used): A bucket, a large measuring cup, a small measuring cup, a wax crayon, *Splash-less* bleach, cotton swabs, and mupirocin ointment.

### Follow-up

In total, 5 parent advisors participated in a small follow-up survey in which they were asked to provide feedback on the MEDiC kit. Overall, the advisors had positive reactions to the kit, finding it more helpful than other materials they had been given in the past for MRSA. In particular, they found the instruction booklet to be very helpful. They also provided some suggestions for further improvement, such as including tips for helping to ease discomfort from MRSA or instruction on how to properly clean the bathtub after bleach baths. This feedback suggests that the kit is promising as a tool for helping families with MRSA decolonization even in its current state, but that additional refinements are likely to be found with use and further assessment.

## Discussion

### Contribution to the Literature

In preparation for the MEDiC study, our randomized controlled trial, we engaged 16 advisors (adolescent patients along with parents) with previous experiences with MRSA infections requiring abscess I&D. We engaged them primarily around the concept of hygiene instructions as well as the procedures for MRSA decolonization with the ultimate goal of preventing recurrent infections. These key stakeholders provided us with the perspective of patients with lived experiences and the feedback necessary to develop an MRSA decolonization toolkit for use in the MEDiC study.

Our advisors identified a number of barriers/pain points regarding the 12 MRSA hygiene and decolonization tasks. From these, we were able to extract 5 major themes (1) existing step-by-step decolonization instructions are not user friendly and do not answer advisors’ common questions; (2) families may not have the proper tools for implementing decolonization and hygiene procedures; (3) some decolonization and hygiene procedures may be out of the control of families; (4) decolonization and hygiene procedures are burdensome; (5) advisors had incorrect assumptions about MRSA despite having experience with it; and (6) bleach has positive and negative connotations. In addition, the advisors were able to brainstorm potential solutions for these identified pain points. Building upon the work of the advisors during the workshop, we designed the MRSA decolonization kit. The kit includes (1) an instruction booklet with step-by-step photographic instructions, (2) a bleach bath and mupirocin swab instructional video, (3) a decolonization and hygiene tracking booklet with MRSA education, (4) a packing slip with a list of all study materials, (5) an MRSA hygiene shower hanger as an easily accessible reminder of the hygiene steps, (6) a hygiene tracking booklet with MRSA education, and (7) decolonization supplies (including a bucket, measuring cups, and bleach) required for the decolonization protocol.

This patient-engaged approach is fairly new to MRSA. We acknowledge that the individual hygiene steps and decolonization procedures themselves are not novel and have been well documented [11,15,16,31,32]. However, this *kit*, which incorporates the feedback of patient partners, is—as far as we can tell—the first documented attempt to create a detailed, user-friendly approach to MRSA home treatment based in part on patient engagement. Furthermore, our study is the first engagement effort to include primarily a pediatric population and their parents regarding the topic of MRSA decolonization. In fact, to our knowledge, this represents one of only a handful of published studies regarding patient engagement on the topic of community-acquired MRSA. For instance, a translational research collaborative in New York City recruited and trained barbers and hair stylists from 9 barbershops as part of community engagement and education efforts to create awareness of the dangers of MRSA infections [33]. That, however, was engagement in the implementation phase rather than our approach, which brought in patient partners in the study design phase. Patients as design partners bring a unique perspective to traditional research projects, which is among the reasons this approach is championed by funding organizations such as the Agency for Healthcare Research and Quality (AHRQ) and the Patient-Centered Outcomes Research Institute [34].

### The Role of Human-Centered Design

HCD is a helpful tool for patient engagement and is especially relevant in instances when product design is performed. A study intervention tool (such as the MEDiC kit) is a product that can be used by individuals within a study. HCD has a rich tradition of working to ensure that products are designed with good *fit* for users—patients and parents in this case [25]. Through the participatory methods in the workshop, the advisors were empowered to begin the design process by thinking through the implementation barriers to be solved by the design and potential solutions to these. Facilitators CM and DL acted as design process experts, guiding the advisors through activities that helped them act as co-designers who set the direction and parameters for the design itself.

HCD analysis is qualitative and, as such, employs many different methods across different projects. For this study, authors CM and DL employed Kolko’s analysis strategy based on the DIKW framework. The DIKW framework itself was developed in the field of computer systems. Criticisms of this framework include, first, that it defines the words used for each stage vaguely or in contrast to preexisting definitions. Second, the visual representations of the framework (particularly as a pyramid) imply that each stage can be reached by filtering the content of the previous stage and that this misrepresents the more complicated relationships between data, information, knowledge, and wisdom. Kolko’s work addresses these criticisms in that he defines the concepts in useful terms (particularly in the context of design), describes the gaps between stages, and proposes methods that employ human reasoning and action while processing content in each stage to jump the next gap [29]. As Kolko explains, this process is not as linear as it might be described or perceived. The movement between stages in DIKW is iterative and based not on procedure but on human judgment. Ultimately, there is an interaction between the content of each DIKW stage, the context of the problem to be solved, and the experience each team member brings to their processing of the content. The end stages of DIKW in particular require making *best guesses* based on what was known previously, what has been previously experienced, and new information gained from the data. This is what the HCD discipline often refers to as *intuition*, but is essentially the connectionist view of cognition, which describes what the human brain does to create new concepts where no clear model exists [35]. Kolko describes this in terms of abductive reasoning, in which one produces plausible conclusions based on related knowledge and experiences and some inference [29]. There is an important role for intuition in design, but this *intuition* or connectionist cognition must be grounded in the data. Kolko’s use of DIKW is helpful for providing a structure for analysis and synthesis, and ensuring intuition is led by the data and applied at appropriate stages in the process.

### The Role of Visual Communication

Visual communication strategies, such as those from sensory marketing, must be undertaken with care. The advisors did comment on this during the discussion of changing bleach to be more appealing. In particular, they discussed that they would not want bleach to be changed in a way that would make it look like something a child would want to drink. They wanted the visual communication aspects signaling bleach’s toxicity to remain. The study team did not intend to repackage bleach but did want to avoid unintended amplification of concerns MEDiC study participants might have about bleach. As discussed above, bleach is a substance with many negative associations that are quite reasonable. Given this, the team wanted the materials and language for the study to avoid adding to the understandable anxiety some potential participants might feel when considering a protocol involving bleach baths, particularly for their children. The goal was not to persuade participants but to ensure that the materials did not act as an additional layer of stress or confusion. Visual communication and messaging can be very powerful tools that can cause harm, such as the tobacco industry targeting underage youth to increase tobacco use [36]. On the contrary, visual communication and messaging have been used to improve health behaviors, as in the case of the truth campaign’s successful antismoking advertisements targeting young people [37]. In the context of this particular research study, though the team was designing materials for a protocol believed to be beneficial, the team was careful to focus on clarity, ease of use, and low stimulation rather than persuasion. The team wanted MEDiC study participants to have the opportunity to participate while fully informed and with as smooth a process as we could provide. The team is hopeful for future opportunities to test and refine the kit within the context of a large study as originally planned.

### Limitations

One limitation is the small sample size of the advisors who participated in the workshop. However, all of the patients and parents had experience with skin infections such as MRSA, and a few had previously or were currently utilizing bleach bath protocols, thus making them highly knowledgeable stakeholders in the problem we were trying to solve. Additionally, the team recruited parents with children ranging from 15 months to 18 years of age and adolescents ranging from 10 to 18 years of age attended the workshop. This allowed for a range of perspectives based on patient age.

Another limitation is that we were unfortunately unable to schedule a second workshop that would have worked for the advisors between completion of the kit and the study start date. Thus, only 1 session was held, leaving open the possibility that subsequent sessions might have identified other barriers or additional refinements to the design of the kit. HCD often preaches a *fail early and often* approach, valuing iterative cycles; what Hassi and Laakso call *thinking by doing* [38]. In this spirit, rather than a longer research process to create the *perfect* kit the first time (and risking being off the mark), the team planned to iteratively engage MEDiC trial participants after they began using the prototype kit to explore additional barriers that may have emerged and to identify additional kit refinements that might be made. Unfortunately, due to recruitment challenges in the MEDiC trial, there were too few active participants to engage meaningfully in this effort. We still feel that the kit would benefit from iterative refinement in future work.

### Conclusions

In this first documented attempt to incorporate pediatric patients and their families as key stakeholders regarding MRSA SSTIs, we engaged advisors in an HCD process to cocreate a toolkit to help participants complete MRSA decolonization and hygiene protocols as part of a comparative effectiveness trial comparing hygiene-only education versus decolonization protocols on infection recurrence. From the perspective of patients with lived experiences, these advisors provided the study team with a better understanding of the potential barriers to completion of study protocols the participants would likely face as well as guidance on the design of the kit. This stakeholder engagement was essential and directly led to the development of an MRSA decolonization toolkit (MEDiC kit), which was implemented in the MEDiC comparative effectiveness trial.

## Appendix

Multimedia Appendix 1 Task analysis grid 2.

Multimedia Appendix 2 Task analysis grid 3.

Multimedia Appendix 3 Task analysis grid 4.

Multimedia Appendix 4 Decolonization instruction booklet.

Multimedia Appendix 5 Decolonization instructional video.

Multimedia Appendix 6 Packing slip.

Multimedia Appendix 7 Decolonization tracking booklet.

Multimedia Appendix 8 Hygiene tracking booklet.

Multimedia Appendix 9 Hygiene shower hanger.

## Abbreviations

AHRQ: Agency for Healthcare Research and Quality
DIKW: data, information, knowledge, wisdom
HCD: human-centered design
I&D: incision and drainage
MEDiC: methicillin-resistant Staphylococcus aureus Eradication and Decolonization in Children
MRSA: methicillin-resistant Staphylococcus aureus
RJ: Research Jam
SSTI: skin and soft tissue infection

## References

[1] Orscheln R, Hunstad D, Fritz S, Loughman J, Mitchell K, Storch E, Gaudreault M, Sellenriek P, Armstrong J, Mardis E, Storch G (2009). Contribution of genetically restricted, methicillin-susceptible strains to the ongoing epidemic of community-acquired Staphylococcus aureus infections. Clin Infect Dis.

[2] Herold BC, Immergluck LC, Maranan MC, Lauderdale DS, Gaskin RE, Boyle-Vavra S, Leitch CD, Daum RS (1998). Community-acquired methicillin-resistant Staphylococcus aureus in children with no identified predisposing risk. J Am Med Assoc.

[3] Hersh AL, Chambers HF, Maselli JH, Gonzales R (2008). National trends in ambulatory visits and antibiotic prescribing for skin and soft-tissue infections. Arch Intern Med.

[4] Hultén KG, Kaplan SL, Gonzalez BE, Hammerman WA, Lamberth LB, Versalovic J, Mason EO (2006). Three-year surveillance of community onset health care-associated staphylococcus aureus infections in children. Pediatr Infect Dis J.

[5] Karamatsu ML, Thorp AW, Brown L (2012). Changes in community-associated methicillin-resistant Staphylococcus aureus skin and soft tissue infections presenting to the pediatric emergency department: comparing 2003 to 2008. Pediatr Emerg Care.

[6] Lautz TB, Raval MV, Barsness KA (2011). Increasing national burden of hospitalizations for skin and soft tissue infections in children. J Pediatr Surg.

[7] Mistry RD (2013). Skin and soft tissue infections. Pediatr Clin North Am.

[8] Moran GJ, Abrahamian FM, Lovecchio F, Talan DA (2013). Acute bacterial skin infections: developments since the 2005 Infectious Diseases Society of America (IDSA) guidelines. J Emerg Med.

[9] Vaska VL, Nimmo GR, Jones M, Grimwood K, Paterson DL (2012). Increases in Australian cutaneous abscess hospitalisations: 1999-2008. Eur J Clin Microbiol Infect Dis.

[10] Mera RM, Suaya JA, Amrine-Madsen H, Hogea CS, Miller LA, Lu EP, Sahm DF, O'Hara P, Acosta CJ (2011). Increasing role of Staphylococcus aureus and community-acquired methicillin-resistant Staphylococcus aureus infections in the United States: a 10-year trend of replacement and expansion. Microb Drug Resist.

[11] Liu C, Bayer A, Cosgrove S, Daum R, Fridkin S, Gorwitz R, Kaplan S, Karchmer A, Levine D, Murray B, Rybak MJ, Talan DA, Chambers HF, Infectious Diseases Society of America (2011). Clinical practice guidelines by the infectious diseases society of America for the treatment of methicillin-resistant Staphylococcus aureus infections in adults and children. Clin Infect Dis.

[12] Sreeramoju P, Porbandarwalla NS, Arango J, Latham K, Dent DL, Stewart RM, Patterson JE (2011). Recurrent skin and soft tissue infections due to methicillin-resistant Staphylococcus aureus requiring operative debridement. Am J Surg.

[13] Williams DJ, Cooper WO, Kaltenbach LA, Dudley JA, Kirschke DL, Jones TF, Arbogast PG, Griffin MR, Creech CB (2011). Comparative effectiveness of antibiotic treatment strategies for pediatric skin and soft-tissue infections. Pediatrics.

[14] Holsenback H, Smith L, Stevenson MD (2012). Cutaneous abscesses in children: epidemiology in the era of methicillin-resistant Staphylococcus aureus in a pediatric emergency department. Pediatr Emerg Care.

[15] Fritz SA, Camins BC, Eisenstein KA, Fritz JM, Epplin EK, Burnham C, Dukes J, Storch GA (2011). Effectiveness of measures to eradicate Staphylococcus aureus carriage in patients with community-associated skin and soft-tissue infections: a randomized trial. Infect Control Hosp Epidemiol.

[16] Fritz SA, Hogan PG, Hayek G, Eisenstein KA, Rodriguez M, Epplin EK, Garbutt J, Fraser VJ (2012). Household versus individual approaches to eradication of community-associated Staphylococcus aureus in children: a randomized trial. Clin Infect Dis.

[17] Immergluck L, Jain S, Ray S, Mayberry R, Satola S, Parker T, Yuan K, Mohammed A, Jerris R (2017). Risk of skin and soft tissue infections among children found to be Staphylococcus aureus MRSA USA300 carriers. West J Emerg Med.

[18] Kumar N, David MZ, Boyle-Vavra S, Sieth J, Daum RS (2015). High Staphylococcus aureus colonization prevalence among patients with skin and soft tissue infections and controls in an urban emergency department. J Clin Microbiol.

[19] Bode LG, Kluytmans JA, Wertheim HF, Bogaers D, Vandenbroucke-Grauls CM, Roosendaal R, Troelstra A, Box AT, Voss A, van der Tweel I, van Belkum A, Verbrugh HA, Vos MC (2010). Preventing surgical-site infections in nasal carriers of Staphylococcus aureus. N Engl J Med.

[20] Laupland KB, Conly JM (2003). Treatment of Staphylococcus aureus colonization and prophylaxis for infection with topical intranasal mupirocin: an evidence-based review. Clin Infect Dis.

[21] Wendt C, Schinke S, Württemberger M, Oberdorfer K, Bock-Hensley O, von Baum H (2007). Value of whole-body washing with chlorhexidine for the eradication of methicillin-resistant Staphylococcus aureus: a randomized, placebo-controlled, double-blind clinical trial. Infect Control Hosp Epidemiol.

[22] Muenks C, Hogan P, Morelli J, Wang J, Thompson R, Fritz S (2015). Compliance, feasibility, and cost of a methicillin-resistant Staphylococcus aureus decolonization protocol. Open Forum Infect Dis.

[23] (2000). ClinicalTrials.

[24] Moore C, Wiehe S, Lynch D, Claxton G, Landman M, Carroll A, Musey P (2020). Methicillin-Resistant Staphylococcus aureus Eradication and Decolonization in Children Study (Part 2): Patient- and Parent-Centered Outcomes of Decolonization. J Participat Med.

[25] Giacomin J (2015). What Is Human Centred Design?. Des J.

[26] Shepherd A (1998). HTA as a framework for task analysis. Ergonomics.

[27] Krishna A (2012). An integrative review of sensory marketing: engaging the senses to affect perception, judgment and behavior. J Consum Psychol.

[28] Ackoff RL (1999). From data to wisdom. Ackoff's Best: His Classic Writings on Management.

[29] Kolko J (2011). Exposing the Magic of Design: A Practitioner's Guide to the Methods & Theory of Synthesis.

[30] (2018). Research Jam.

[31] Cluzet VC, Gerber JS, Metlay JP, Nachamkin I, Zaoutis TE, Davis MF, Julian KG, Linkin DR, Coffin SE, Margolis DJ, Hollander JE, Bilker WB, Han X, Mistry RD, Gavin LJ, Tolomeo P, Wise JA, Wheeler MK, Hu B, Fishman NO, Royer D, Lautenbach E, CDC Prevention Epicenters Program (2016). The effect of total household decolonization on clearance of colonization with methicillin-resistant Staphylococcus aureus. Infect Control Hosp Epidemiol.

[32] Huang SS, Singh R, McKinnell JA, Park S, Gombosev A, Eells SJ, Gillen DL, Kim D, Rashid S, Macias-Gil R, Bolaris MA, Tjoa T, Cao C, Hong SS, Lequieu J, Cui E, Chang J, He J, Evans K, Peterson E, Simpson G, Robinson P, Choi C, Bailey CC, Leo JD, Amin A, Goldmann D, Jernigan JA, Platt R, Septimus E, Weinstein RA, Hayden MK, Miller LG (2019). Decolonization to reduce postdischarge infection risk among MRSA carriers. N Engl J Med.

[33] Leinberger-Jabari A, Kost RG, D'Orazio B, Burgess R, Khalida C, Tsang A, Mitchell D, Tomasz A, de Lencastre H, de la Gandara MP, Evering TH, Holder T, Coller BS, Tobin JN (2016). From the bench to the barbershop: community engagement to raise awareness about community-acquired methicillin-resistant and hepatitis C virus infection. Prog Community Health Partnersh.

[34] Concannon TW, Fuster M, Saunders T, Patel K, Wong JB, Leslie LK, Lau J (2014). A systematic review of stakeholder engagement in comparative effectiveness and patient-centered outcomes research. J Gen Intern Med.

[35] Coyne RD (1990). Design reasoning without explanations. AI Mag.

[36] Perry CL (1999). The tobacco industry and underage youth smoking: tobacco industry documents from the Minnesota litigation. Arch Pediatr Adolesc Med.

[37] Farrelly MC, Nonnemaker J, Davis KC, Hussin A (2009). The Influence of the National truth campaign on smoking initiation. Am J Prev Med.

[38] Hassi L, Laakso M, Karjalainen T, Koria M, Salimäki M (2011). Making sense of design thinking. IDBM Papers Volume 1.

