# Supplementary figures and images for: Methicillin-Resistant Staphylococcus aureus Eradication and Decolonization in Children Study (Part 1): Development of a Decolonization Toolkit With Patient and Parent Advisors
Source: J Particip Med. 2020 May 20;12(2):e14974. doi: 10.2196/14974 (PMC7434080; doi:10.2196/14974)

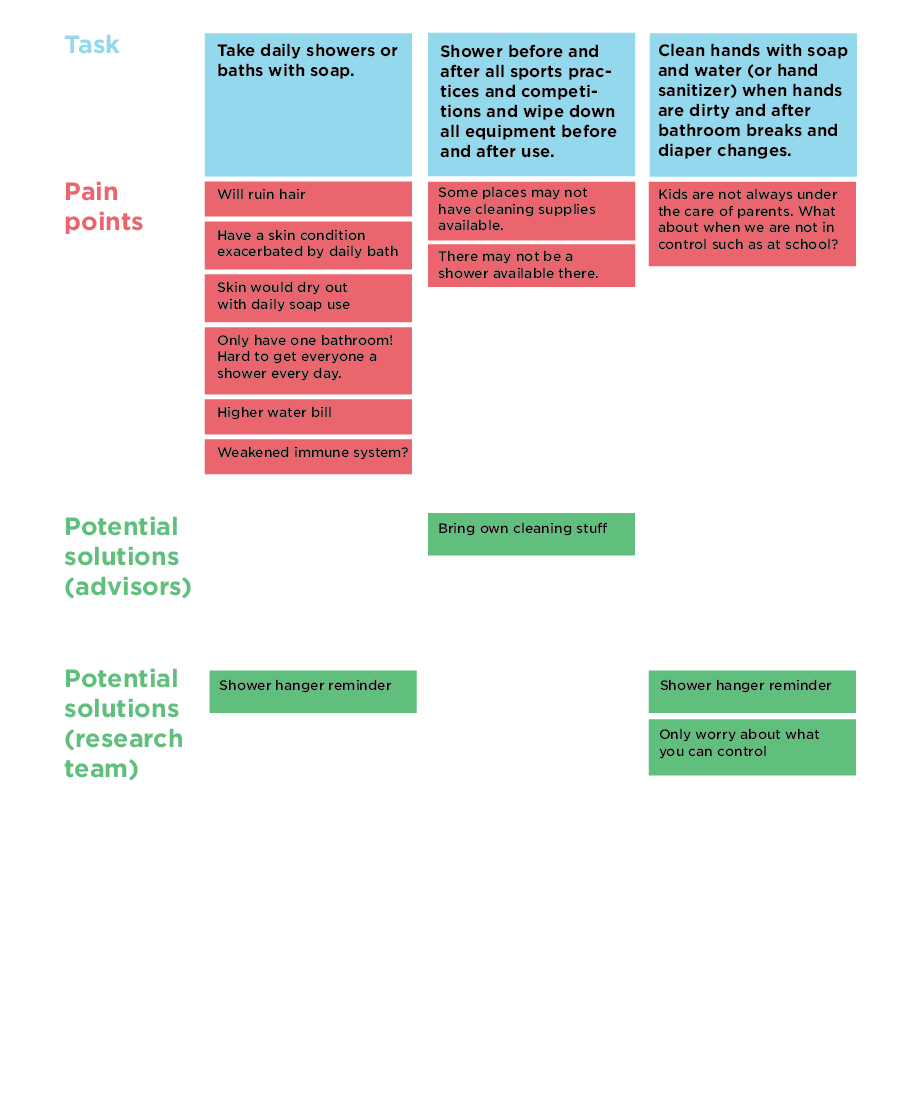

Supplement: Multimedia Appendix 1 [file jopm_v12i2e14974_app1.png]

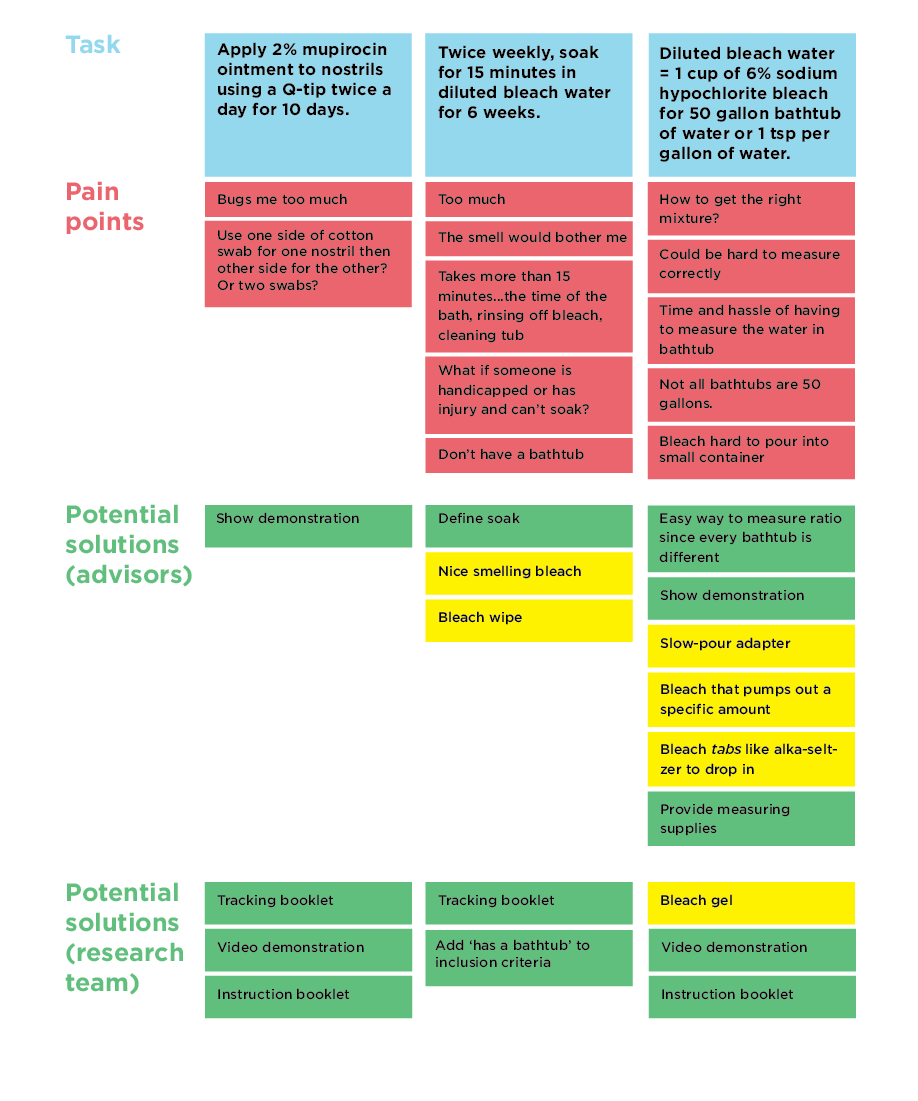

Supplement: Multimedia Appendix 2 [file jopm_v12i2e14974_app2.png]

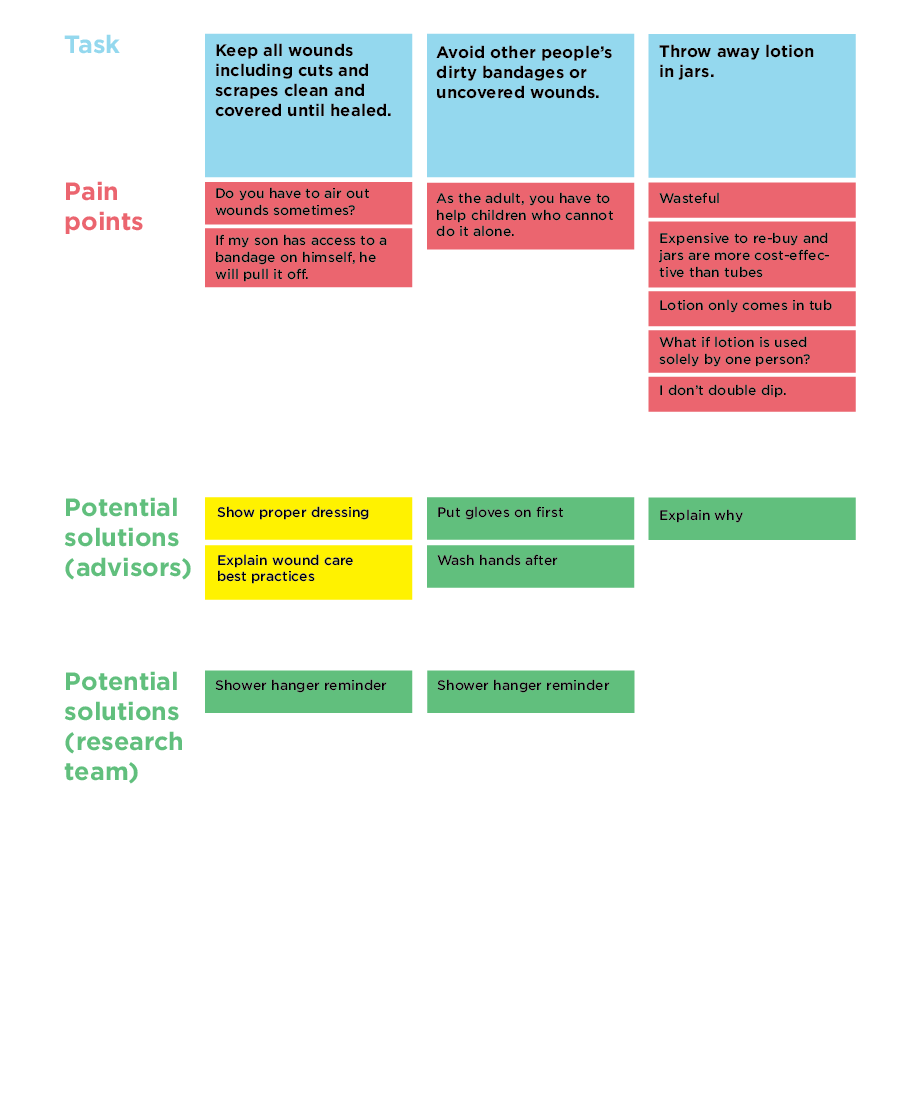

Supplement: Multimedia Appendix 3 [file jopm_v12i2e14974_app3.png]
