# Supplementary material for: Methicillin-Resistant Staphylococcus aureus Eradication and Decolonization in Children Study (Part 1): Development of a Decolonization Toolkit With Patient and Parent Advisors
Source: J Particip Med. 2020 May 20;12(2):e14974. doi: 10.2196/14974 (PMC7434080; doi:10.2196/14974)
Supplement: Multimedia Appendix 4 [file jopm_v12i2e14974_app4.pdf]

Bleach Bath & Mupirocin Swab Instructions

MRSA Eradication and Decolonization in Children

MEDiC Kit

This book takes you step by step through setting up your bleach baths and completing your mupirocin swabs for the study. These steps use the supplies you should have gotten in the mail.

Do your best to take a bleach bath two times a week for six weeks and do the mupirocin swabs two times a day for ten days. Be sure to keep track of the tasks you complete in your Study Tracking Book (the little blue book). If you miss something, that's okay. Only track the tasks you complete.

If you'd like to see a video showing the steps in this book in action, visit https://youtu.be/rrGi2NCvV9Y.

Bleach Baths

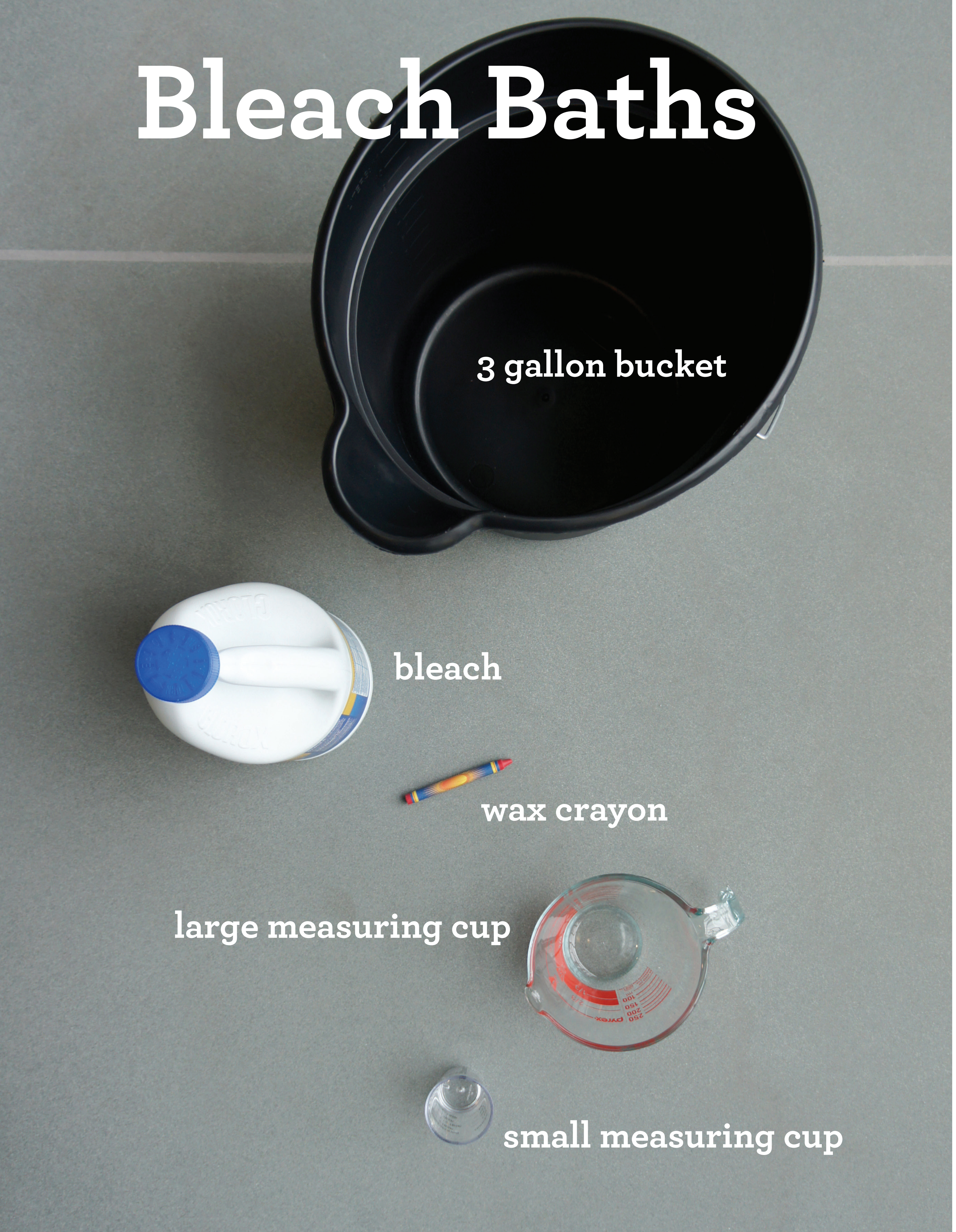

15 minute soaks twice per week for 6 weeks

1 Find out how many buckets of water you need to fill your tub:

Plug your tub so it won't drain. Fill the bucket with water (up to the 3 gallon mark) and pour it out into the tub. Count 1.

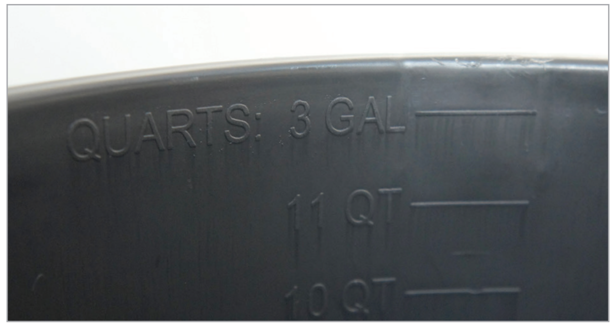

Repeat and count how many times you empty the bucket into the tub until the tub is filled to the level you would usually use for a bath for you or your child. Write down or remember the number of buckets.

2 Mark the water level:

Use the wax marking pencil to make a mark on the side of the tub where the top of the water is.

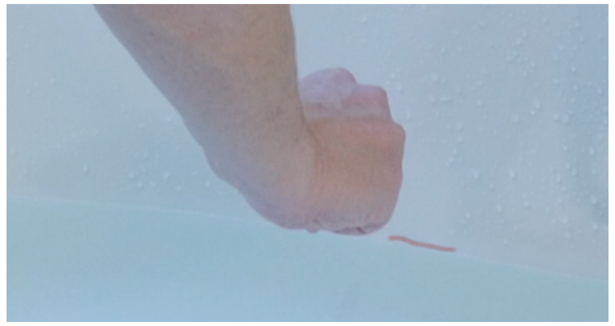

Leave this mark there. From now on, you can fill your tub up to this mark instead of counting buckets again.

3 Measure out the bleach:

In general, 2 teaspoons of bleach will need to be added for every 3 gallons of water. Remember, the bucket we sent you holds 3 gallons so you can just add 2 teaspoons of bleach for every bucket you used.

Here's a handy bleach measurement chart if you need it:

WATER	BLEACH
1 1/2 gallon<br>*(1/2 of a bucket)*	1 teaspoon
3 gallons<br>*(1 bucket)*	2 teaspoons
6 gallons<br>*(2 buckets)*	4 teaspoons
12 gallons<br>*(4 buckets)*	8 teaspoons
19 gallons<br>*(6 1/3 buckets)*	1/4 cup
25 gallons<br>*(8 1/3 buckets)*	1/3 cup
40 gallons ½<br>*(13 1/3 buckets)*	1/2 cup
50 gallons<br>*(16 2/3 buckets)*	2/3 cup

Measure out the correct amount of bleach using the large measuring cup or the small measuring cup (depending on how much you need). Pour this into your tub of water.

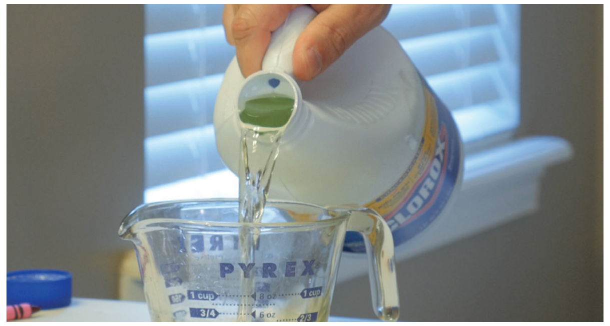

4 Soak for 15 minutes then mark down that you took a bleach bath in your Study Tracking Book.

That's it! This process can be used for infant tubs as well. Just start back at Step 1.

Mupirocin Swabs

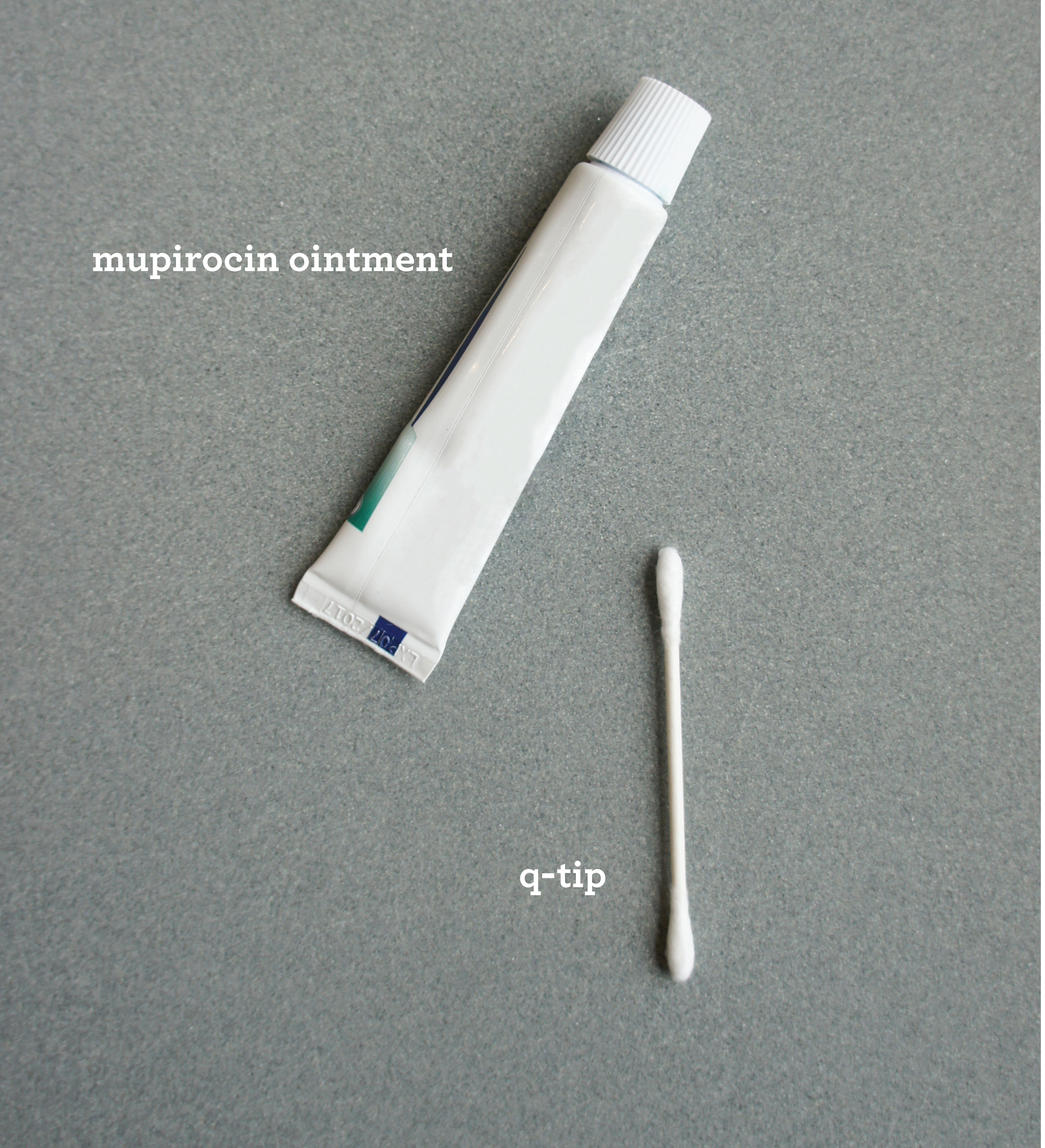

Twice per day for 10 days.

1 Get the ointment ready:

Take one of the double sided q-tips and squeeze a tiny amount of mupirocin ointment on each side of the q-tip. Close the mupirocin.

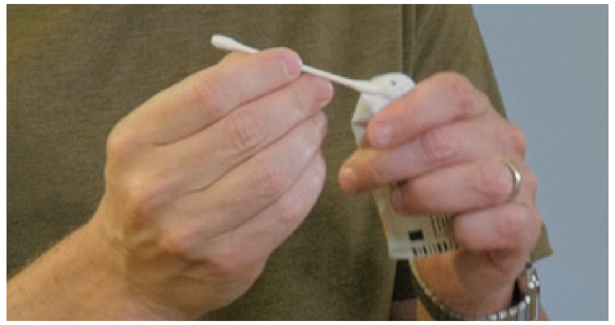

2 Put the ointment on:

Use one side of the q-tip to spread the mupi-rocin inside one nostril. Then use *the other side* for the other nostril.

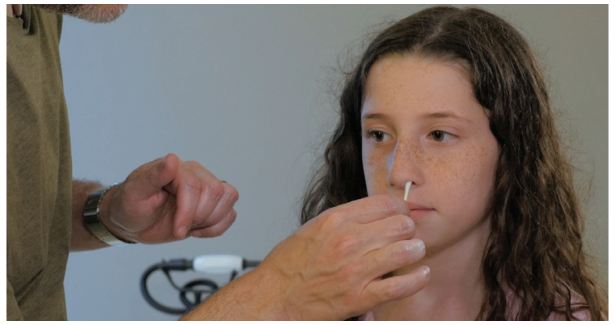

You only need to put the q-tip just inside the nostril. You do not need to go very deep.

3 Clean up:

Throw the q-tip away and wash your hands with warm water and soap.

4 Check the box for one of today's mupirocin swabs on the calendar in your Study Tracking Book.

That's it! Do this twice a day for 10 days.

MRSA Eradication and Decolonization in Children

MEDiC Kit

INDIANA UNIVERSITY

The MEDiC Kit was developed as a part of the MRSA Eradication and Decolonization in Children (MEDiC) study conducted by Dr. Paul Musey, Jr, Dr. Matthew Landman, and Dr. Aaron Carrol. The study team collaborated with the Indiana CTSI's Research Jam team. These materials were developed with guidance by families with experience in MRSA. The goal of the kit is to guide families step by step through best practices for MRSA decolonization using bleach baths and mupirocin swabs. These materials were developed through grant funding to principal investigator Aaron Carroll from (AHRQ - 1R24HS022434-01).
