# Supplementary material for: Methicillin-Resistant Staphylococcus aureus Eradication and Decolonization in Children Study (Part 1): Development of a Decolonization Toolkit With Patient and Parent Advisors
Source: J Particip Med. 2020 May 20;12(2):e14974. doi: 10.2196/14974 (PMC7434080; doi:10.2196/14974)
Supplement: Multimedia Appendix 6 [file jopm_v12i2e14974_app6.pdf]

MRSA Eradication and Decolonization in Children
MEDiC Kit

Supplies you should've received:

bleach

This should have (or will) come in a shipment by itself. Remember to keep bleach (and all cleaning supplies) away from children.

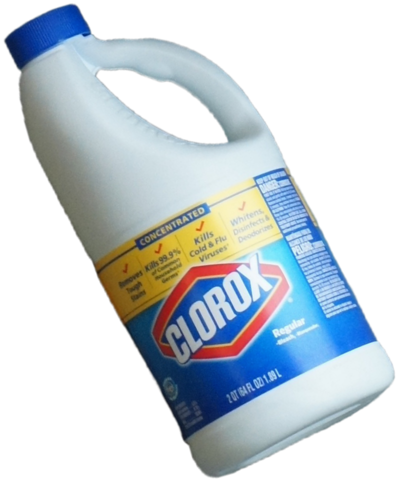

mupirocin ointment

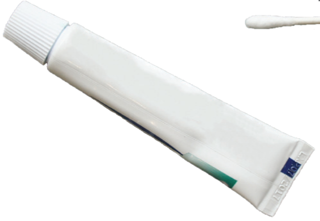

q-tips

wax crayon

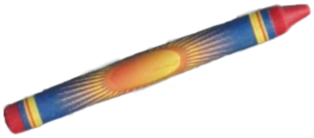

small measuring cup

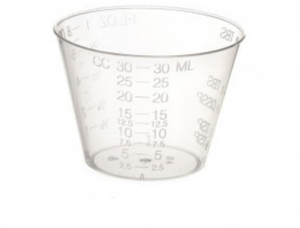

large measuring cup

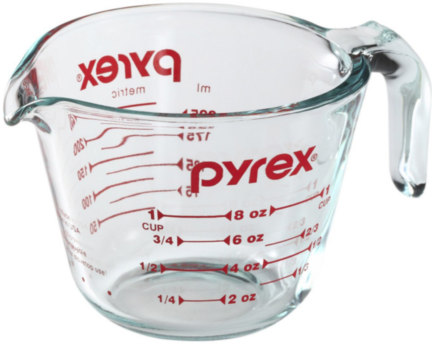

tracking book

This study tracking book belongs to

....

(first name only please)

Please start the study on a Sunday.

This book will help you track the study tasks you complete (mupirocin swabs and bleach baths). Do your best to complete all the study tasks, but if you miss something, that's okay. Only track the tasks you complete. The book also includes hygiene steps you can take to help avoid MRSA as well as space to track new boils/abscesses and missed school or work for up to a year.

3 gallon bucket

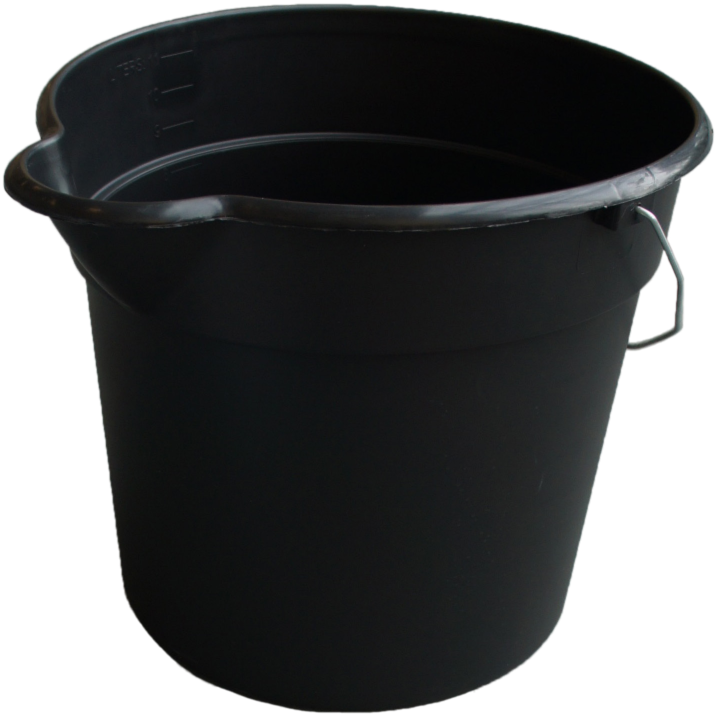

instruction book

Bleach Bath & Mupirocin Swab Instructions

MRSA Eradication and Decolonization in Children
MEDiC Kit

shower hanger

Remember:

Take daily showers or baths and wash hands throughout the day.

Keep wounds covered and avoid the wounds of others.

Don't share items like clothes or personal hygiene tools.

Do laundry weekly.

Keep sports/exercise equipment clean.
