# Supplementary material for: Methicillin-Resistant Staphylococcus aureus Eradication and Decolonization in Children Study (Part 1): Development of a Decolonization Toolkit With Patient and Parent Advisors
Source: J Particip Med. 2020 May 20;12(2):e14974. doi: 10.2196/14974 (PMC7434080; doi:10.2196/14974)
Supplement: Multimedia Appendix 7 [file jopm_v12i2e14974_app7.pdf]

This study tracking book belongs to

. . . .

(first name only please)

Please start the study on a Sunday.

This book will help you track the study tasks you complete (mupirocin swabs and bleach baths). Do your best to complete all the study tasks, but if you miss something, that's okay. Only track the tasks you complete. The book also includes hygiene steps you can take to help avoid MRSA as well as space to track new boils/abscesses and missed school or work for up to a year.

what is MRSA exactly?

The basics

MRSA stands for Methicillin-resistant Staphylococcus aureus. Basically, MRSA is a type of staph bacteria that can't be killed by the antibiotics that usually work for other types of staph bacteria. Many people have staph bacteria on their bodies (especially in their noses), but only about 2% of the population have MRSA bacteria and some people who do have MRSA on their bodies will not get an infection. An infection happens when there are so many of the MRSA bacteria that the body can't keep them under control. People who are more likely to get a MRSA infection are people who have spent time in hospitals or nursing homes, who have weakened immune systems, who are involved in contact sports, and small children (especially if they go to daycare).

How it spreads

MRSA is spread by touching a person who has it on their skin or by touching objects that carry MRSA. That's why it's really important for people with MRSA to keep their skin clean, to clean objects they use, and not to share objects that are likely to carry MRSA.

Frequently asked questions

1 IF I HAVE MRSA, HOW CONTAGIOUS AM I?

If you have symptoms of an active MRSA infection (pain, swelling, boil, abscess, wound draining pus, etc.), you are contageous and you could spread MRSA because the infected area contains many MRSA germs. Keep the area clean and keep the wound covered. If you do not have an active infection, there is still a small risk of spreading MRSA to close contacts such as family members.

2 I HAVE A MRSA INFECTION. SHOULD I GO TO SCHOOL OR WORK?

You should ONLY go to school or work IF...
- the infection can be completely covered with a bandage or dressing.
- all wound drainage can be contained all day with only one dressing change at work or school.
- you are willing and able to follow good hand washing and personal hygiene.

Until open wounds are healed...
- do not use whirlpools or swimming pools.
- do not participate in contact sports or other activities in which bandages or dressings may fall off
- do not use playgrounds or bounce houses.

bleach baths & nose swabs

1. Fill in the dates in the blue boxes starting with today (Sunday). Don't forget to turn the page.
2. Do 2 nose swabs with mupirocin (morning & evening) each day for 10 days. Check the box for each swab you complete.
3. Each week, take 2 bleach baths. Write "bath 1" on the day you take the 1st one and "bath 2" on the day you take the 2nd.
4. Write any side effects you have on the calendar.

	Sun.	Mon.	Tues.	Wed.	Thurs.	Fri.	Sat.
**week one**	☐ morning<br>☐ evening	☐ morning<br>☐ evening	☐ morning<br>☐ evening	☐ morning<br>☐ evening	☐ morning<br>☐ evening	☐ morning<br>☐ evening	☐ morning<br>☐ evening
**week two**	☐ morning<br>☐ evening	☐ morning<br>☐ evening	☐ morning<br>☐ evening				
**week three**							

	Sun.	Mon.	Tues.	Wed.	Thurs.	Fri.	Sat.
week four							

	Sun.	Mon.	Tues.	Wed.	Thurs.	Fri.	Sat.
week five							

	Sun.	Mon.	Tues.	Wed.	Thurs.	Fri.	Sat.
week six							

hygiene instructions

Here are some simple ways that you and your family can help prevent the recurrence of MRSA outbreaks. We realize that some things may be out of your control, but the more of these you can do, the better your chances of staying outbreak free.

Keep skin clean

- Take daily showers or baths with soap.
- Clean hands with soap and water or with hand sanitizers when hands are dirty and after each bathroom break or diaper change.

Avoid wounds

- Keep all wounds including cuts and scrapes clean and covered until healed.
- Avoid the dirty bandages or uncovered wounds of other people. If you must help a child with their wounds, wash your hands with soap or use hand sanitizer afterward.

Don't share items that could be contaminated

- Don't share bath towels, wash cloths, clothing, tooth-brushes, or razors within the family or with friends.

  We realize it may not be practical for everyone to have a separate hand towel in the bathroom so one solution may be to use paper towels.

- Throw away and do not share lotions in jars.

  The lotion can be easily contaminated with MRSA when someone puts their hand in the jar. Try to use pump or squeeze bottles instead. If you absolutely must use a jar, start off with a fresh one and never put your hand inside the jar. Instead, use a clean spoon or other tool to get the lotion out. Don't double dip! Clean the tool after every use.

Do laundry regularly

- Wash all towels, wash cloths, sleepwear, underwear, and linens that have been used in hot water with laundry detergent once weekly and dry with hot air in a dryer.

Keep sports and exercise equipment clean

- Encourage athletes or health club members to shower before and after all practices and competitions, and wipe down equipment surfaces before and after use.
- Uniforms and practice jerseys should be washed after each game or practice. Sports equipment should be washed/cleaned weekly.

ugh, another boil!

For 1 year, please track new boils/abscesses and how they are treated. This will help us see if the study tasks have helped you have less outbreaks.

1 date ______________
- ☐ drainage procedure
- ☐ antibiotic __________

2 date ______________
- ☐ drainage procedure
- ☐ antibiotic __________

3 date ______________
- ☐ drainage procedure
- ☐ antibiotic __________

4 date ______________
- ☐ drainage procedure
- ☐ antibiotic __________

5 date ______________
- ☐ drainage procedure
- ☐ antibiotic __________

6 date ______________
- ☐ drainage procedure
- ☐ antibiotic __________

7 date ______________
- ☐ drainage procedure
- ☐ antibiotic __________

8 date ______________
- ☐ drainage procedure
- ☐ antibiotic __________

9 date ______________
- ☐ drainage procedure
- ☐ antibiotic __________

10 date ______________
- ☐ drainage procedure
- ☐ antibiotic __________

11 date ______________
- ☐ drainage procedure
- ☐ antibiotic __________

12 date ______________
- ☐ drainage procedure
- ☐ antibiotic __________

13 date ______________
- ☐ drainage procedure
- ☐ antibiotic __________

14 date ______________
- ☐ drainage procedure
- ☐ antibiotic __________

15 date ______________
- ☐ drainage procedure
- ☐ antibiotic __________

16 date ______________
- ☐ drainage procedure
- ☐ antibiotic __________

17 date ______________
- ☐ drainage procedure
- ☐ antibiotic __________

18 date ______________
- ☐ drainage procedure
- ☐ antibiotic __________

19 date ______________
- ☐ drainage procedure
- ☐ antibiotic __________

20 date ______________
- ☐ drainage procedure
- ☐ antibiotic __________

21 date ______________
- ☐ drainage procedure
- ☐ antibiotic __________

22 date ______________
- ☐ drainage procedure
- ☐ antibiotic __________

no school/work today

For 1 year, please track any days of missed work or school because of your outbreaks. This will help us see if the study tasks have helped you miss less school or work because of outreaks.

1 date ______________
- ☐ missed school
- ☐ missed work

2 date ______________
- ☐ missed school
- ☐ missed work

3 date ______________
- ☐ missed school
- ☐ missed work

4 date ______________
- ☐ missed school
- ☐ missed work

5 date ______________
- ☐ missed school
- ☐ missed work

6 date ______________
- ☐ missed school
- ☐ missed work

7 date ______________
- ☐ missed school
- ☐ missed work

8 date ______________
- ☐ missed school
- ☐ missed work

9 date ______________
- ☐ missed school
- ☐ missed work

10 date ______________
- ☐ missed school
- ☐ missed work

11 date ______________
- ☐ missed school
- ☐ missed work

12 date ______________
- ☐ missed school
- ☐ missed work

13 date ______________
- ☐ missed school
- ☐ missed work

14 date ______________
- ☐ missed school
- ☐ missed work

15 date ______________
- ☐ missed school
- ☐ missed work

16 date ______________
- ☐ missed school
- ☐ missed work

17 date ______________
- ☐ missed school
- ☐ missed work

18 date ______________
- ☐ missed school
- ☐ missed work

19 date ______________
- ☐ missed school
- ☐ missed work

20 date ______________
- ☐ missed school
- ☐ missed work

21 date ______________
- ☐ missed school
- ☐ missed work

22 date ______________
- ☐ missed school
- ☐ missed work

notes

This page is for any notes you want to make about the study. Some things you could write are details about side effects you have during the study, any problems you have with the study tasks, any questions you have for the study team, or anything else you'd like the study team to know about your experience.

MRSA Eradication and Decolonization in Children

MEDiC Kit

INDIANA UNIVERSITY

The MEDiC Kit was developed as a part of the MRSA Eradication and Decolonization in Children (MEDiC) study conducted by Dr. Paul Musey, Jr, Dr. Matthew Landman, and Dr. Aaron Carrol. The study team collaborated with the Indiana CTSI's Research Jam team. These materials were developed with guidance by families with experience in MRSA. The goal of the kit is to guide families step by step through best practices for MRSA decolonization using bleach baths and mupirocin swabs. These materials were developed through grant funding to principal investigator Aaron Carroll from (AHRQ - 1R24HS022434-01).
