# Supplementary material for: Methicillin-Resistant Staphylococcus aureus Eradication and Decolonization in Children Study (Part 1): Development of a Decolonization Toolkit With Patient and Parent Advisors
Source: J Particip Med. 2020 May 20;12(2):e14974. doi: 10.2196/14974 (PMC7434080; doi:10.2196/14974)
Supplement: Multimedia Appendix 9 [file jopm_v12i2e14974_app9.pdf]

Remember:

Take daily showers or baths and wash hands throughout the day.

Keep wounds covered and avoid the wounds of others.

Don’t share items like clothes or personal hygiene tools.

Do laundry weekly.

Keep sports/exercise equipment clean.

MRSA Eradication and Decolonization in Children

MEDiC Kit

INDIANA UNIVERSITY

The MEDiC Kit was developed as a part of the MRSA Eradication and Decolonization in Children (MEDiC) study conducted by Dr. Paul Musey, Jr, Dr. Matthew Landman, and Dr. Aaron Carrol. The study team collaborated with the Indiana CTSI's Research Jam team. These materials were developed with guidance by families with experience in MRSA. The goal of the kit is to guide families step by step through best practices for MRSA decolonization using bleach baths and mupirocin swabs. These materials were developed through grant funding to principal investigator Aaron Carroll from (AHRQ - 1R24HS022434-01).
